# Adolescent, Pregnant, and HIV-Infected: Risk of Adverse Pregnancy and Perinatal Outcomes in Young Women from Southern Mozambique

**DOI:** 10.3390/jcm10081564

**Published:** 2021-04-08

**Authors:** Clara Pons-Duran, Aina Casellas, Azucena Bardají, Anifa Valá, Esperança Sevene, Llorenç Quintó, Eusebio Macete, Clara Menéndez, Raquel González

**Affiliations:** 1 ISGlobal, Hospital Clínic-Universitat de Barcelona, 08036 Barcelona, Spain; aina.casellas@isglobal.org (A.C.); azucena.bardaji@isglobal.org (A.B.); llorenc.quinto@isglobal.org (L.Q.); clara.menendez@isglobal.org (C.M.); raquel.gonzalez@isglobal.org (R.G.); 2 Consorcio de Investigación Biomédica en Red de Epidemiología y Salud Pública (CIBERESP), 28029 Madrid, Spain; 3 Departament de Fonaments Clínics, Facultat de Medicina, Universitat de Barcelona (UB), Casanova 143, 08036 Barcelona, Spain; 4 Manhiça Health Research Center (CISM), Manhiça 1929, Mozambique; anifa.vala@manhica.net (A.V.); esperanca.sevene@manhica.net (E.S.); eusebio.macete@manhica.net (E.M.); 5 Department of Physiological Science, Clinical Pharmacology, Faculty of Medicine, Eduardo Mondlane University, Maputo 3453, Mozambique

**Keywords:** maternal health, pregnancy, adolescent’s health, HIV infection, sub-Saharan Africa

## Abstract

Sub-Saharan Africa concentrates the burden of HIV and the highest adolescent fertility rates. However, there is limited information about the impact of the interaction between adolescence and HIV infection on maternal health in the region. Data collected prospectively from three clinical trials conducted between 2003 and 2014 were analysed to evaluate the association between age, HIV infection, and their interaction, with the risk of maternal morbidity and adverse pregnancy and perinatal outcomes in women from southern Mozambique. Logistic regression and negative binomial models were used. A total of 2352 women were included in the analyses; 31% were adolescents (≤19 years) and 29% HIV-infected women. The effect of age on maternal morbidity and pregnancy and perinatal adverse outcomes was not modified by HIV status. Adolescence was associated with an increased incidence of hospital admissions (IRR 0.55, 95%CI 0.37–0.80 for women 20–24 years; IRR 0.60, 95%CI 0.42–0.85 for women >25 years compared to adolescents; *p*-value < 0.01) and outpatient visits (IRR 0.86, 95%CI 0.71–1.04; IRR 0.76, 95%CI 0.63–0.92; *p*-value = 0.02), and an increased likelihood of having a small-for-gestational age newborn (OR 0.50, 95%CI 0.38–0.65; OR 0.43, 95%CI 0.34–0.56; *p*-value < 0.001), a low birthweight (OR 0.40, 95%CI 0.27–0.59; OR 0.37, 95%CI 0.26–0.53; *p*-value <0.001) and a premature birth (OR 0.42, 95%CI 0.24–0.72; OR 0.51, 95%CI 0.32–0.82; *p*-value < 0.01). Adolescence was associated with an increased risk of poor morbidity, pregnancy and perinatal outcomes, irrespective of HIV infection. In addition to provision of a specific maternity care package for this vulnerable group interventions are imperative to prevent adolescent pregnancy.

## 1. Introduction

It is estimated that approximately 12 million girls between 15 and 19 years old and 777,000 girls under 15 years gave birth in low- and middle-income countries, the vast majority in Asia and Africa [1,2]. Adolescent and young girls worldwide, especially in low-resource settings, suffer from a disproportionate burden of disease due to preventable and treatable health problems, including sexual, reproductive, and maternal health problems [3]. Young women’s health, and especially that of adolescent girls, constitutes thus a global health priority [3,4]. It is known that adolescent mothers have higher risk than other age groups to die or suffer from disability as a result of pregnancy, while their newborns present higher risk of being born with low birth weight (LBW), prematurity or asphyxia, which contributes to an increase in infant mortality and psychomotor development problems [5,6,7,8,9,10,11].

Sub-Saharan Africa (SSA) also concentrates the highest burden of Human Immunodeficiency Virus (HIV) infection. In 2019, 19.1 million women of 15 years or more were living with the infection in the world, 80% of them in SSA [12,13]. In 2018, more than 900 thousand pregnancies happened among HIV-infected women in the 23 most affected countries [13]. Moreover, in 2019 there were 350,000 new infections among adolescent girls and young women (under 25 years) in SSA [12], which is a major issue of concern in this region where more than 50% of pregnancies take place during adolescence [14].

Most of the HIV-infected women living in SSA are diagnosed during antenatal care (ANC) services through prevention of mother-to-child transmission programs (PMTCT). Since 2013, the World Health Organization (WHO) recommends lifelong antiretroviral treatment (ART) (Option B+) for HIV-infected pregnant and post-partum women [15]. In 2018, almost 82% of women living with HIV received ART [12]. However, adherence varies across countries and it is influenced by demographic and health indicators such as mental health, stigma, marital status, or age [16,17]. Importantly, adolescent girls present a reduced rate of ART uptake, adherence to PMTCT strategies, and consequently, are at an increased risk of mother-to-child transmission [18,19,20].

Adolescence, pregnancy, and HIV infection may constitute a dangerous overlap, particularly in SSA. Nevertheless, there is limited information about the interactions of the three conditions and their concomitant impact on maternal health [11,21]. Of note, a recent scoping review about the experiences of adolescent mothers living with HIV in SSA, concluded that information about pregnancy and perinatal outcomes of this population group was almost non-existent [22].

The objective of this study was to investigate the presence and effect of an interaction between HIV infection and age on maternal morbidity and the prevalence of adverse pregnancy and perinatal outcomes among women living in a semi-rural area from SSA.

## 2. Materials and Methods

### 2.1. Study Design and Data Sources

This study was a secondary analysis of data obtained prospectively from three clinical trials conducted in the district of Manhiça, southern Mozambique, between 2003 and 2014 [23,24,25]. The trials evaluated the safety and efficacy of antimalarial drugs as Intermittent Preventive Treatment of malaria in pregnancy (IPTp). The first trial was a double-blind randomized placebo-controlled trial that evaluated two doses of sulfadoxine-pyrimethamine (SP) for IPTp versus placebo among women, regardless of their HIV status [23]. The second study was an open label randomized clinical trial among HIV-uninfected pregnant women where the efficacy and safety of two doses of IPTp-mefloquine (MQ) and two doses of IPTp-SP were compared [24]. Finally, the third study was a randomized double-blind clinical trial that included HIV-infected women on cotrimoxazole prophylaxis (CTXp) and compared the efficacy and safety of three doses of IPTp-MQ with three-dose IPTp-placebo [25]. The summary of the results of the three clinical trials can be found in the Supplementary material (Text S1).

### 2.2. Study Population

The study population was comprised of pregnant women enrolled in the three abovementioned clinical trials. The inclusion criteria for the trials were the same with the exception of HIV infection: (a) ≤28 weeks of gestation when attending the ANC clinic for the first time during the current pregnancy, (b) being residents in the study area, and (c) agreeing to give birth in one of the maternity wards of the study area. Women were followed up until one month after the end of pregnancy.

The population subgroup of interest in the present study was adolescents. Following WHO definition, adolescence was defined as being between 10 and 19 years old [26]. Young women aged 20 to 24 years and adult women ≥25 years were used as comparison groups.

### 2.3. Study Area

All data collected belong to women from the Manhiça district, southern Mozambique. Manhiça is a semi-rural area of around 200,000 inhabitants [27]. The participants were recruited at the ANC clinics of the Manhiça District Hospital and the Maragra Health Centre between 2003 and 2014 [23,24,25]. In 2012, the prevalence of HIV infection at the community level was estimated to be nearly of 40% in the adult population and 29% among pregnant women [28,29].

At the time the studies were conducted, PMTCT relied on nevirapine (NVP) prophylaxis during pregnancy (2003–2005) or on daily monotherapy with zidovudine (AZT) from 14 weeks of gestation (2010–2014). In addition, between 2010 and 2014, maternal prophylaxis was combined with antiretroviral (ARV) drugs during labour and up to one week postpartum (single dose nevirapine (sd-NVP) and daily AZT plus lamivudine (3TC)), together with the administration of daily NVP to the infant, from birth until one week after weaning. ART was recommended when CD4+T cell count decreased to levels <350 and/or when the woman was in 3 or 4 HIV/AIDS WHO clinical stage. Roll out of ARV drugs for ART in the study area started in 2005 and these were delivered to pregnant women at the monthly ANC visits.

### 2.4. Study Variables

The study outcomes were maternal morbidity and adverse pregnancy and perinatal outcomes. Maternal morbidity was defined by the frequency of hospital admissions and outpatient visits during pregnancy of study participants from trial’s enrolment until study completion. The adverse pregnancy outcomes analysed were miscarriage—defined as a foetal loss before completing the 20th weeks of gestation; and stillbirth—a loss from week 20 until delivery. Adverse perinatal outcomes included were LBW—birth weight less than 2500 g; small for gestational age newborns (SGA)—defined as live newborns with a birth weight below the 10th percentile for their gestational age; and preterm babies defined as born before week 37 of gestation. Of note, stillbirths could be also considered perinatal events depending on the definition used.

The main independent variables of the analyses were maternal HIV status, which was categorized into infected and uninfected, and age group, adolescents (≤19 years), young women (20–24 years) and adult women (≥25 years).

Additional variables of interest used as covariates or potential confounding factors were trial arm, literacy, gestational age at recruitment, mid-upper arm circumference (MUAC), and anaemia at recruitment [8].

### 2.5. Data Cleaning and Analysis

Databases of the three trials were pooled in a single database and data cleaning was carried out prior to the analyses. Three hundred and thirty-seven observations were dropped because of missing data on at least one of the covariates or the HIV status. After this, the proportion of HIV- infected women and the proportion of adolescents remained constant. The variables with the highest proportion of missing data were HIV status (5.8% of women had an unknown status), and MUAC at baseline (7.7% of the sample had no MUAC value). The analyses of LBW had a lower number of observations due to the exclusion of women who had a miscarriage, and of cases with missed information on birth weight. In addition, the analyses of SGA and premature births had 307 fewer observations due to missing data of gestational age at delivery.

Data were described using frequencies, means and standard deviations for discrete and continuous variables, respectively. Logistic regression models with penalized likelihood were used to explore an interaction effect between HIV and age group regarding each dichotomous outcome [30]. The penalized likelihood method is used when data have complete or quasi-complete separation, or the prevalence/coverage of the outcome is low like in this study. For count outcomes, negative binomial models were run as they are suitable for this type of outcome measures. These models were estimated taking each subject follow-up time into account. For each outcome, univariable and multivariable models were computed. All covariates were forced to be kept in the multivariable models to allow for comparability across them. The interaction between HIV status and age group was tested in the multivariable models. Gravidity was not included in the models to avoid confounding since it was correlated with age [7,8]. Nevertheless, stratified analyses by age were performed to assess the effect of gravidity. The significance level was set at 0.05. Data analyses were performed using the Stata statistical program version 16 (Stata Corporation, College Station, TX, USA) [31].

## 3. Results

Data from 2352 pregnant women contributed to the analyses. The characteristics of the study population stratified by age category are shown in Table 1. Of the total sample, 31% were adolescents and 29% were HIV-infected women. Fourteen per cent of the pregnant adolescent girls were HIV-infected.

In one of the included trials, 84% of HIV-infected women reported having taken nevirapine for PMTCT at delivery [32]. In the trial on HIV-infected pregnant women, more than 90% of the participants reported having taken ARV drugs for PMTCT during pregnancy, while 75% of all enrolled women were adherent to 80% or more of the expected CTXp doses [25].

Univariable models of the maternal morbidity outcomes displayed significant associations with both HIV-infection and age group (Table 2). Compared with young and adult women, adolescents had a higher risk of hospital admissions during pregnancy (IRR 0.60 95%CI 0.41–0.87 and IRR 0.67 95%CI 0.49–0.92, respectively, *p*-value < 0.01). Although no significant difference was observed between adolescents and young women, age was found to decrease the risk of outpatient visits (IRR 0.86 95%CI 0.71–1.05 for young women and IRR 0.75 95%CI 0.63–0.90 for adults, *p*-value < 0.01, with adolescence as reference category). HIV-infection was also associated with increased risk of hospital admissions during pregnancy (IRR 1.39 95%CI 1.04–1.87, *p*-value = 0.03). Regarding adverse pregnancy outcomes, the probability of having a miscarriage or a stillbirth was not significantly associated with maternal age in the univariable models (Table 3). HIV infection was associated with an increased likelihood of having a stillbirth (OR 1.84 95%CI 1.14–2.97, *p*-value = 0.01). Regarding perinatal outcomes, adolescence was significantly associated with an increased likelihood of having a LBW, a SGA, and a premature newborn (*p*-value < 0.001 in all cases) (Table 4).

**Figure 1:**
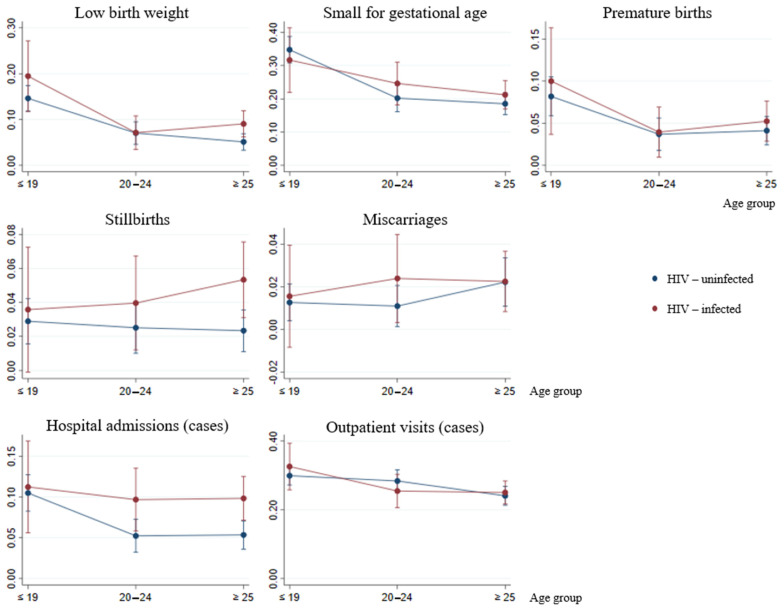
Age and HIV infection interaction: predictive margins with 95% CIs, by study outcome.

The interaction between HIV infection and maternal age was not statistically significant in any multivariable analysis. Figure 1 shows the predictive margins of the tested interactions between HIV status and age group, by study outcome. The effect of age over morbidity or pregnancy and perinatal adverse outcomes was not modified by HIV status.

In the multivariable regression models, adolescent girls had a significantly increased incidence of hospital admissions and outpatient visits than young and adult women (IRR 0.55 95%CI 0.37–0.80, and IRR 0.60 95%CI 0.42–0.85, respectively, *p*-value < 0.01, for hospital admissions; IRR 0.86 95%CI 0.71–1.04 and IRR 0.76 95%CI 0.63–0.92, *p*-value = 0.02, for outpatient visits) (Table 2). Prevalence rates of stillbirths and miscarriages were not associated with maternal age (Table 3). Young women and adults were less likely than adolescent girls to have a LBW newborn (OR 0.40 95%CI 0.27–0.59 and OR 0.37 95%CI 0.26–0.53, respectively, *p*-value < 0.001) (Table 4). With regard to SGA newborns, adolescent girls accounted for a higher risk compared to adults and young women (OR 0.50 95%CI 0.38–0.65 for women 20–24 years, and OR 0.43 95%CI 0.34–0.56 for adult women, with adolescents as reference category, *p*-value < 0.001) (Table 4). The same applies to premature newborns; young (OR 0.42 95%CI 0.24–0.72) and adult women (OR 0.51 95%CI 0.32–0.82) had a significantly lower probability of preterm birth (*p*-value < 0.01) than adolescent women (Table 4).

In the multivariable models, HIV infection was significantly associated with a lower risk of outpatient visits during pregnancy (IRR 0.83 95%CI 0.69–1.00, *p*-value < 0.05) (Table 2) and with an increased prevalence of LBW babies (OR 1.52 95%CI 1.07–2.16, *p*-value = 0.02) (Table 4).

The stratified analyses by age group, which included gravidity among the covariates, showed that multigravidae adolescents were less likely to have a LBW baby (OR 0.44 95%CI 0.22–0.72, *p*-value < 0.01) and a SGA newborn (OR 0.44 95%CI 0.29–0.68, *p*-value < 0.001) than primigravid adolescents. In addition, multigravidae ≥25 years old had a lower risk of having a miscarriage (OR 0.10 95%CI 0.02–0.61, *p*-value = 0.01) or a stillbirth (OR 0.17 95%CI 0.05–0.60, *p*-value < 0.01) than primigravidae of the same age (Supplementary Materials, Table S1).

## 4. Discussion

To our knowledge, this is the first analysis evaluating the potential interaction between maternal age and HIV status on maternal morbidity and adverse pregnancy and perinatal outcomes in women from SSA. Hospital admissions and outpatient visits during pregnancy, LBW, SGA, and premature births were more frequent among adolescents than in young women and adults, independently of HIV status.

These results are in line with studies where adolescent pregnancies have been associated with increased risk of maternal morbidity and adverse outcomes such as eclampsia, LBW, preterm birth and puerperal endometritis [5,6,7,8,9,10,11]. These associations have been found to be caused by biological factors related to age, rather than socio-demographic and behavioural determinants [11]. The associations of maternal morbidity and adverse pregnancy and perinatal outcomes with mother’s age found in our analysis were not modified by HIV status.

The fact that most pregnant adolescent girls are primigravidae could partially explain the associations observed between young age and impaired maternal health, which could not be completely independent of gravidity. Associations between primigravity and increased risks of adverse maternal health outcomes have been extensively described and they are partially explained by women’s biological immaturity in their first pregnancy [7,8]. However, our findings suggest that this association also depends on maternal age. Gravidity was associated with LBW and SGA prevalence only among adolescents, and with stillbirths and miscarriages among women ≥ 25 years of age. Similarly, significant associations between young age and preterm birth and LBW have been reported in a study including primigravidae women only [6].

When considering the generalizability of the findings, it is worth mentioning that the study participants were all women who attended monthly ANC visits. Since pregnancy during adolescence is a taboo in many cultures, it is likely that a significant proportion of adolescent girls do not attend the ANC services. Thus, the observed figures, although robust and significant, could be underestimating the maternal health risks associated with adolescent’s pregnancies. On the other hand, participants in clinical trials might be different in terms of socio-economic background than the general population, especially if acceptance rates are low and attendance to health services poor. However, this is unlikely in the context of the present study since the proportion of women that accepted to participate in the study trials was generally over 97%. Additionally, the Manhiça district has a long history of health surveillance and epidemiological research with a high engagement of the community [27].

The lack of a significant interaction between maternal age and HIV infection, suggests that adolescence is not affecting disproportionately maternal health of HIV-infected girls compared to that of those HIV-uninfected. Our findings indicate that HIV infection is related to the same maternal health risks at all ages. This finding could be due to the fact that many HIV-infected adolescent girls may be recently infected and do not present complications. However, HIV-infected adolescents had increased morbidity and frequency of poor pregnancy-related outcomes than HIV-infected pregnant adults. Therefore, maternal health programs should target adolescent girls, regardless of their HIV status.

The increased prevalence of LBW newborns among HIV-infected women in the multivariable model is consistent with previous studies [33,34,35,36]. Contrary to this finding, the incidence of outpatient visits was increased in HIV-uninfected women. Potential explanations for this association include a high efficacy of CTXp [37], which was taken by women participating in the clinical trial that only enrolled HIV-infected pregnant women, and a lower attendance to ANC by HIV-infected women due to stigma.

The association between HIV infection and adverse pregnancy and perinatal outcomes remains controversial. First, not all available studies found significant associations between HIV infection and poor maternal health outcomes [38]. Then, some authors suggest that the success of strategies such as PMTCT programs could be reverting the negative effects of HIV over outcomes like LBW [39]. Moreover, there is a growing body of literature suggesting that the use of several ART regimens is associated with higher prevalence rates of adverse perinatal and pregnancy events [40,41]. At the time the first clinical trial was conducted in 2003, PMTCT of HIV relied on NVP prophylaxis, and CTXp was only given to pregnant women with CD4+T counts below 350 cells/µL. From all HIV-infected participating women, 84% reported taking NVP at childbirth onset [32]. On the other hand, in the trial conducted from 2010 to 2014, all participants received CTXp and over 90% of them adhered to ART or PMTCT. The present study was not adjusted by ART, PMTCT, and CTXp adherence, which constitutes an important limitation for the interpretation, thus, a possible association between HIV and other study outcomes rather than LBW cannot be discarded.

Several study limitations related to the data and analysis should be acknowledged. First, the data sources are old and from different years, when HIV treatment and prevention guidelines, HIV knowledge and adherence to ANC were different. This caveat makes more complex the interpretation of the results. Secondly, the observational nature of the analyses makes impossible to infer causality, therefore only associations can be observed and hypothesis generated. Finally, the multivariable models created are exploratory in nature, only allowing to assess the modification of the relationship between the study outcomes and age in the presence of HIV infection. The models are not generalizable.

Despite the limitations, the strengths of this study make it relevant for informing policy and research on adolescents’ health and HIV. The principal strength of the present study is the robust analytical methodology that was used. This was an individual-patient data pooled analysis of three large clinical studies performed in the same SSA setting, enrolling more than 2000 pregnant women. Importantly, to our knowledge, this study is the first evaluating the interaction between age and HIV infection with pregnancy and perinatal outcomes in this vulnerable group of women from SSA.

In conclusion, adolescent pregnancies are associated with an increased likelihood of poor maternal health outcomes similarly among HIV-infected and uninfected pregnant women. Global efforts are devoted to prevent adolescent pregnancies through adolescent-friendly services that increase access of girls to contraceptive methods, such as information and counselling services on reproductive health, and prevention, detection and response programs on sexual and other forms of gender-based violence [2,3,4]. Additionally, there is an urgent need of strategies targeting adolescent girls once they are already pregnant, such as the provision of an adapted maternity care package for this vulnerable group that tackles their special needs, or strategies to empower young mothers to overcome stigma. Additional research on the concomitant impact of global epidemics such as HIV on adolescent’s maternal health from SSA is needed.

## Figures and Tables

**Table 1:** Characteristics of study population, by age group.

Variable		Age in Years (Categories) N = 2352			*p*-Value
		≤19 *n* = 729	20–24 *n* = 628	≥25 *n* = 995	
Demographic and clinical data					
HIV status ^1^	Infected	101 (14)	195 (31)	397 (40)	<0.001
	Uninfected	628 (86)	433 (69)	598 (60)	
Gravidity ^1^	Primigravidae	550 (75)	89 (14)	16 (2)	<0.001
	Multigravidas	179 (25)	539 (86)	979 (98)	
Weight at baseline (in kg) ^2^		58.1 (7.0)	59.4 (7.8)	62.3 (10.2)	<0.001
Gestational age at recruitment ^2^		21.1 (4.6)	21.3 (5.0)	21.7 (4.9)	0.09
Anaemia (<11 g/dl Hb) ^1^	Yes	401 (55)	331 (53)	544 (55)	0.66
	No	328 (45)	297 (48)	451 (45)	
MUAC at baseline ^1^	Normal	698 (96)	616 (98)	981 (99)	<0.001
	Lower or equal to 22	31 (4)	12 (2)	14 (1)	
Literacy ^1^	Literate	592 (81)	425 (68)	548 (55)	<0.001
	Illiterate	137 (19)	203 (32)	447 (45)	
Data origin					
Trial ^1^	Menéndez et al. 2008	216 (30)	209 (33)	256 (26)	<0.001
	González et al. 2014 (I)	451 (62)	276 (44)	415 (42)	
	González et al. 2014 (II)	62 (9)	143 (23)	324 (33)	
Study arm ^1^	Placebo	132 (18)	171 (27)	295 (30)	<0.001
	MQ	326 (45)	262 (42)	438 (44)	
	SP	271 (37)	195 (31)	262 (26)	

^1^ n (Column percentage)/Chi-squared test *p*-value ^2^ Arithmetic Mean (SD)/ANOVA *p*-value.

**Table 2:** Univariable and multivariable analyses: maternal morbidity outcomes.

Variable		Study Outcome			
		Hospital Admissions during Pregnancy *n* = 2352		Outpatient Visits during Pregnancy *n* = 2352	
Univariable analyses					
		IRR (95% CI)	*p*-value	IRR (95% CI)	*p*-value
HIV status ^1^		1.39 (1.04–1.87)	0.03	0.91 (0.77–1.08)	0.28
Age in years	≤19	1	<0.01	1	<0.001
	20–24	0.60 (0.41–0.87)		0.86 (0.71–1.05)	
	≥25	0.67 (0.49–0.92)		0.75 (0.63–0.90)	
Multivariable analysis					
		IRR (95% CI)	*p*-value	IRR (95% CI)	*p*-value
HIV status ^1^		1.28 (0.90–1.81)	0.17	0.83 (0.69–1.00)	<0.05
Age in years	≤19	1	<0.01	1	0.02
	20–24	0.55 (0.37–0.80)		0.86 (0.71–1.04)	
	≥25	0.60 (0.42–0.85)		0.76 (0.63–0.92)	

^1^ IRR for Positive vs. Negative Note: Multivariable analyses adjusted for study arm, gestational age at recruitment, MUAC at baseline, literacy and anaemia.

**Table 3:** Univariable and multivariable analyses: adverse pregnancy outcomes.

Variable		Study Outcome			
		Miscarriages *n* = 2352		Stillbirths *n* = 2352	
Univariable analyses					
		OR (95% CI)	*p*-value	OR (95% CI)	*p*-value
HIV status ^1^		1.38 (0.70–2.71)	0.35	1.84 (1.14–2.97)	0.01
Age in years	≤19	1	0.30	1	0.73
	20–24	1.16 (0.45–3.03)		0.99 (0.52–1.89)	
	≥25	1.78 (0.80–3.99)		1.20 (0.69–2.10)	
Multivariable analysis					
		OR (95% CI)	*p*-value	OR (95% CI)	*p*-value
HIV status ^1^		0.93 (0.39–2.25)	0.87	1.74 (0.99–3.04)	0.05
Age in years	≤19	1	0.25	1	0.78
	20–24	1.10 (0.40–3.03)		0.92 (0.47–1.79)	
	≥25	1.91 (0.78–4.67)		1.13 (0.62–2.06)	

^1^ OR for Positive vs. Negative Note: Multivariable analyses adjusted for study arm, gestational age at recruitment, MUAC at baseline, literacy and anaemia.

**Table 4:** Univariable and multivariable analyses: adverse perinatal outcomes.

Variable		Study Outcome					
		Low Birth Weight *n* = 2299		Small for Gestational Age *n* = 2045		Premature Births *n* = 2045	
Univariable analyses							
		OR (95% CI)	*p*-value	OR (95% CI)	*p*-value	OR (95% CI)	*p*-value
HIV status ^1^		1.09 (0.80–1.48)	0.58	0.93 (0.74–1.16)	0.50	0.99 (0.64–1.51)	0.95
Age in years	≤19	1	<0.001	1	<0.001	1	<0.001
	20–24	0.42 (0.29–0.61)		0.52 (0.40–0.68)		0.42 (0.24–0.71)	
	≥25	0.39 (0.28–0.55)		0.46 (0.37–0.59)		0.52 (0.34–0.79)	
Multivariable analysis							
		OR (95% CI)	*p*-value	OR (95% CI)	*p*-value	OR (95% CI)	*p*-value
HIV status ^1^		1.52 (1.07–2.16)	0.02	1.10 (0.85–1.42)	0.47	1.34 (0.83–2.16)	0.23
Age in years	≤19	1	<0.001	1	<0.001	1	<0.01
	20–24	0.40 (0.27–0.59)		0.50 (0.38–0.65)		0.42 (0.24–0.72)	
	≥25	0.37 (0.26–0.53)		0.43 (0.34–0.56)		0.51 (0.32–0.82)	

^1^ OR for Positive vs. Negative Note: Multivariable analyses adjusted for study arm, gestational age at recruitment, MUAC at baseline, literacy, and anaemia.

## Data Availability

The data presented in this study are available on request from TIMNET and MiPPAD trials consortiums (clara.menendez@isglobal.org). The data are not publicly available due to privacy and ethics reasons.

## Supplementary Materials

The following are available online at https://www.mdpi.com/article/10.3390/jcm10081564/s1, Table S1: Sensitivity multivariable analyses by age group: associations between gravidity and the study outcomes, Text S1: Summary of the three clinical trials included in the analyses.

## References

[1] Darroch J.E., Woog V., Bankoleand A., Ashford L.S. (2016). Adding It Up: Costs and Benefits of Meeting the Contraceptive Needs of Adolescents. https://www.guttmacher.org/report/adding-it-meeting-contraceptive-needs-of-adolescents.

[2] World Health Organization (2018). Adolescents: Health Risks and Solutions. https://www.who.int/news-room/fact-sheets/detail/adolescents-health-risks-and-solutions.

[3] United Nations (2015). The Global Strategy for Women’s, Children’s and Adolescents’ Health (2016–2030). https://www.who.int/life-course/partners/global-strategy/globalstrategyreport2016-2030-lowres.pdf.

[4] WHO (2015). Core Competencies in Adolescent Health and Development for Primary Care Providers: Including a Tool to Assess the Adolescent Health And Development Component in Pre-Service Education of Health-Care Providers. https://www.who.int/maternal_child_adolescent/documents/core_competencies/en/.

[5] Ganchimeg T., Ota E., Morisaki N., Laopaiboon M., Lumbiganon P., Zhang J., Yamdamsuren B., Temmerman M., Say L., Tunçalp Ö. (2014). Pregnancy and childbirth outcomes among adolescent mothers: A World Health Organization multicountry study. BJOG Int. J. Obstet. Gynaecol..

[6] Ganchimeg T., Mori R., Ota E., Koyanagi A., Gilmour S., Shibuya K., Torloni M.R., Betran A.P., Seuc A., Vogel J. (2013). Maternal and perinatal outcomes among nulliparous adolescents in low- and mid-dle-income countries: A multi-country study. BJOG.

[7] Kozuki N., Lee A.C.C., Silveira M.F., Sania A., Vogel J.P., Adair L., Barros F., Caulfield L.E., Christian P., Fawzi W. (2013). The associations of parity and maternal age with small-for-gestational-age, preterm, and neonatal and infant mortality: A meta-analysis. BMC Public Health.

[8] Mombo-Ngoma G., Mackanga J.R., González R., Ouedraogo S., Kakolwa M.A., Manego R.Z., Basra A., Rupérez M., Cot M., Kabanywany A.M. (2016). Young adolescent girls are at high risk for adverse pregnancy outcomes in sub-Saharan Africa: An observational multicountry study. BMJ Open.

[9] Grønvik T., Sandøy I.F. (2018). Complications associated with adolescent childbearing in Sub-Saharan Africa: A systematic literature review and meta-analysis. PLoS ONE.

[10] Chen X.K., Wen S.W., Fleming N., Demissie K., Rhoads G.G., Walker M. (2007). Teenage pregnancy and adverse birth outcomes: A large population based retro-spective cohort study. Int. J. Epidemiol..

[11] Fraser A.M., Brockert J.E., Ward R. (1995). Association of Young Maternal Age with Adverse Reproductive Outcomes. N. Engl. J. Med..

[12] UNAIDS (2020). AIDSinfo. https://aidsinfo.unaids.org/.

[13] UNAIDS (2019). Start Free Stay Free AIDS Free. https://www.unaids.org/sites/default/files/media_asset/20190722_UNAIDS_SFSFAF_2019_en.pdf.

[14] Callahan T., Modi S., Swanson J., Ng’Eno B., Broyles L.N. (2017). Pregnant adolescents living with HIV: What we know, what we need to know, where we need to go. J. Int. AIDS Soc..

[15] WHO (2016). Consolidated Guidelines on the Use of Antiretroviral Drugs for Treating and Preventing HIV Infection: Rec-Ommendations for a Public Health Approach.

[16] Psaros C., Smit J.A., Mosery N., Bennett K., Coleman J.N., Bangsberg D.R., Safren A.S. (2020). PMTCT Adherence in Pregnant South African Women: The Role of Depression, Social Support, Stigma, and Structural Barriers to Care. Ann. Behav. Med..

[17] Mukosha M., Chiyesu G., Vwalika B. (2020). Adherence to antiretroviral therapy among HIV infected pregnant women in public health sectors: A pilot of Chilenje level one Hospital Lusaka, Zambia. Pan Afr. Med. J..

[18] Fatti G., Shaikh N., Eley B., Jackson D., Grimwood A. (2014). Adolescent and young pregnant women at increased risk of mother-to-child transmission of HIV and poorer maternal and infant health outcomes: A cohort study at public facilities in the Nelson Mandela Bay Metropolitan district, Eastern Cape, South Africa. S. Afr. Med. J..

[19] Ronen K., McGrath C.J., Langat A.C., Kinuthia J., Omolo D., Singa B., Katana A.K., Ng’Ang’ A.L.W., John-Stewart G. (2017). Gaps in Adolescent Engagement in Antenatal Care and Prevention of Moth-er-to-Child HIV Transmission Services in Kenya. J. Acquir. Immune Defic. Synd..

[20] Ramraj T., Jackson D., Dinh T.-H., Olorunju S., Lombard C., Sherman G., Puren A., Ramokolo V., Noveve N., Singh Y. (2018). Adolescent Access to Care and Risk of Early Mother-to-Child HIV Transmission. J. Adolesc. Health.

[21] Groves A.K., Maman S., Stankard P.H., Gebrekristos L.T., Amon J.J., Moodley D. (2018). Addressing the unique needs of adolescent mothers in the fight against HIV. J. Int. AIDS Soc..

[22] Toska E., Laurenzi C.A., Roberts K.J., Cluver L., Sherr L. (2020). Adolescent mothers affected by HIV and their children: A scoping review of evidence and experiences from sub-Saharan Africa. Glob. Public Health.

[23] Menéndez C., Bardají A., Sigauque B., Romagosa C., Sanz S., Serra-Casas E., Macete E., Berenguera A., David C., Dobaño C. (2008). A Randomized Placebo-Controlled Trial of Intermittent Preventive Treatment in Pregnant Women in the Context of Insecticide Treated Nets Delivered through the Antenatal Clinic. PLoS ONE.

[24] González R., Mombo-Ngoma G., Ouedraogo S., Kakolwa M.A., Abdulla S., Accrombessi M., Aponte J.J., Akerey-Diop D., Basra A., Briand V. (2014). Intermittent Preventive Treatment of Malaria in Pregnancy with Mefloquine in HIV-Negative Women: A Multicentre Randomized Controlled Trial. PLoS Med..

[25] González R., Desai M., Macete E., Ouma P., Kakolwa M.A., Abdulla S., Aponte J.J., Bulo H., Kabanywanyi A.M., Katana A. (2014). Intermittent Preventive Treatment of Malaria in Pregnancy with Mefloquine in HIV-Infected Women Receiving Cotrimoxazole Prophylaxis: A Multicenter Randomized Placebo-Controlled Trial. PLoS Med..

[26] WHO (2020). Adolescent Pregnancy. https://www.who.int/news-room/fact-sheets/detail/adolescent-pregnancy.

[27] Sacoor C., Nhacolo A., Nhalungo D., Aponte J.J., Bassat Q., Augusto O., Mandomando I., Sacarlal J., Lauchande N., Sigaúque B. (2012). Profile: Manhica Health Research Centre (Manhica HDSS). Int. J. Epidemiol..

[28] González R., Munguambe K., Aponte J., Bavo C., Nhalungo D., Macete E., Alonso P., Menéndez C., Naniche D. (2012). High HIV prevalence in a southern semi-rural area of Mozambique: A community-based survey. HIV Med..

[29] González R., Augusto O.J., Munguambe K., Pierrat C., Pedro E.N., Sacoor C., De Lazzari E., Aponte J.J., Macete E., Alonso P.L. (2015). HIV Incidence and Spatial Clustering in a Rural Area of Southern Mozambique. PLoS ONE.

[30] Firth D. (1993). Bias reduction of maximum likelihood estimates. Biometrika.

[31] StataCorp (2019). Stata Statistical Software: Release 16.

[32] Naniche D., Lahuerta M., Bardaji A., Sigauque B., Romagosa C., Berenguera A., Mandomando I., David C., Sanz S., Aponte J. (2008). Mother-to-child transmission of HIV-1: Association with malaria prevention, anaemia and placental malaria. HIV Med..

[33] Rollins N.C., Coovadia H.M., Bland R.M., Coutsoudis A., Bennish M.L., Patel D., Newell M.-L. (2007). Pregnancy Outcomes in HIV-Infected and Uninfected Women in Rural and Urban South Africa. JAIDS J. Acquir. Immune Defic. Syndr..

[34] Santosa W.B., Staines-Urias E., Tshivuila-Matala C.O., Norris S.A., Hemelaar J. (2019). Perinatal outcomes associated with maternal HIV and an-tiretroviral therapy in pregnancies with accurate gestational age in South Africa. AIDS.

[35] Xiao P.-L., Zhou Y.-B., Chen Y., Yang M.-X., Song X.-X., Shi Y., Jiang Q.-W. (2015). Association between maternal HIV infection and low birth weight and prematurity: A meta-analysis of cohort studies. BMC Pregnancy Childbirth.

[36] González R., Rupérez M., Sevene E., Vala A., Maculuve S., Bulo H., Nhacolo A., Mayor A., Aponte J.J., Macete E. (2017). Effects of HIV infection on maternal and neonatal health in southern Mozambique: A prospective cohort study after a decade of antiretroviral drugs roll out. PLoS ONE.

[37] Moraleda C., de Deus N., Serna-Bolea C., Renom M., Quintó L., Macete E., Menéndez C., Naniche D. (2014). Impact of HIV exposure on health outcomes in HIV-negative infants born to HIV-positive mothers in Sub-Saharan Africa. J. Acquir. Immune Defic. Syndr..

[38] Goldman G., Budhram S., Budhram S. (2020). A retrospective cohort study comparing pregnancy outcomes and neonatal char-acteristics between HIV-infected and HIV-non-infected mothers. S. Afr. Med. J..

[39] Twabi H.S., Manda S.O., Small D.S. (2020). Assessing the effects of maternal HIV infection on pregnancy outcomes using cross-sectional data in Malawi. BMC Public Health.

[40] Theron G., Brummel S., Fairlie L., Pinilla M., McCarthy K., Owor M., Chinula L., Makanani B., Violari A., Moodley D. (2020). Pregnancy Outcomes of Women Conceiving on Antiretroviral Therapy (ART) Compared to Those Commenced on ART During Pregnancy. Clin. Infect. Dis..

[41] Tshivuila-Matala C.O., Honeyman S., Nesbitt C., Kirtley S., Kennedy S.H., Hemelaar J. (2020). Adverse perinatal outcomes associated with antiretroviral therapy regimens: Systematic review and network meta-analysis. AIDS.

