# Supplementary material for: Adolescent, Pregnant, and HIV-Infected: Risk of Adverse Pregnancy and Perinatal Outcomes in Young Women from Southern Mozambique
Source: J Clin Med. 2021 Apr 8;10(8):1564. doi: 10.3390/jcm10081564 (PMC8068130; doi:10.3390/jcm10081564)
Supplement: Supplementary file 1 [file jcm-10-01564-s001.pdf]

<u>Text S1. Summary of the three clinical trials included in the analyses</u>

<u>Trial 1</u>

A randomized placebo-controlled trial of intermittent preventive treatment in pregnant women in the context of insecticide treated nets delivered through the antenatal clinic

doi: 10.1371/journal.pone.0001934

Background: Current recommendations to prevent malaria in African pregnant women rely on insecticide treated nets (ITNs) and intermittent preventive treatment (IPTp). However, there is no information on the safety and efficacy of their combined use.

Methods and findings: 1030 pregnant Mozambican women of all gravidities received a long-lasting ITN during antenatal clinic (ANC) visits and, irrespective of HIV status, were enrolled in a randomised, double blind, placebo-controlled trial, to assess the safety and efficacy of 2-dose sulphadoxine-pyrimethamine (SP). The main outcome was the reduction in low birth weight. Two-dose SP was safe and well tolerated, but was not associated with reductions in anaemia prevalence at delivery (RR, 0.92 [95% CI, 0.79-1.08]), low birth weight (RR, 0.99 [95% CI, 0.70-1.39]), or overall placental infection (p = 0.964). However, the SP group showed a 40% reduction (95% CI, 7.40-61.20]; p = 0.020) in the incidence of clinical malaria during pregnancy, and reductions in the prevalence of peripheral parasitaemia (7.10% vs 15.15%) (p<0.001), and of actively infected placentas (7.04% vs 13.60%) (p = 0.002). There was a reduction in severe anaemia at delivery of borderline statistical significance (p = 0.055). These effects were not modified by gravidity or HIV status. Reported ITN's use was more than 90% in both groups.

Conclusions: Two-dose SP was associated with a reduction in some indicators, but these were not translated to significant improvement in other maternal or birth outcomes. The use of ITNs during pregnancy may reduce the need to administer IPTp. ITNs should be part of the ANC package in sub-Saharan Africa.

Trial registration: ClinicalTrials.gov NCT00209781.

Trial 2

Intermittent preventive treatment of malaria in pregnancy with mefloquine in HIV-negative women: a multicentre randomized controlled trial

doi: 10.1371/journal.pmed.1001733

Background: Intermittent preventive treatment in pregnancy (IPTp) with sulfadoxine-pyrimethamine (SP) is recommended by WHO to prevent malaria in African pregnant women. The spread of SP parasite resistance has raised concerns regarding long-term use for IPT. Mefloquine (MQ) is the most promising of available alternatives to SP based on safety profile, long half-life, and high efficacy in Africa. We evaluated the safety and efficacy of MQ for IPTp compared to those of SP in HIV-negative women.

Methods and findings: A total of 4,749 pregnant women were enrolled in an open-label randomized clinical trial conducted in Benin, Gabon, Mozambique, and Tanzania comparing two-dose MQ or SP for IPTp and MQ tolerability of two different regimens. The study arms were: (1) SP, (2) single dose MQ (15 mg/kg), and (3) split-dose MQ in the context of long-lasting insecticide treated nets. There was no difference on low birth weight prevalence (primary study outcome) between groups (360/2,778 [13.0%]) for MQ group and 177/1,398 (12.7%) for SP group; risk ratio [RR], 1.02 (95% CI 0.86-1.22; p=0.80 in the ITT analysis). Women receiving MQ had reduced risks of parasitemia (63/1,372 [4.6%] in the SP group and 88/2,737 [3.2%] in the MQ group; RR, 0.70 [95% CI 0.51-0.96]; p=0.03) and anemia at delivery (609/1,380 [44.1%] in the SP group and 1,110/2743 [40.5%] in the MQ group; RR, 0.92 [95% CI 0.85-0.99]; p=0.03), and reduced incidence of clinical malaria (96/551.8 malaria episodes person/year [PYAR] in the SP group and 130/1,103.2 episodes PYAR in the MQ group; RR, 0.67 [95% CI 0.52-0.88]; p=0.004) and all-cause outpatient attendances during pregnancy (850/557.8 outpatients visits PYAR in the SP group and 1,480/1,110.1 visits PYAR in the MQ group; RR, 0.86 [0.78-0.95]; p=0.003). There were no differences in the prevalence of placental infection and adverse pregnancy outcomes between groups. Tolerability was poorer in the two MQ groups compared to SP. The most frequently reported related adverse events were dizziness (ranging from 33.9% to 35.5% after dose 1; and 16.0% to 20.8% after dose 2) and vomiting (30.2% to 31.7%, after dose 1 and 15.3% to 17.4% after dose 2) with similar proportions in the full and split MQ arms. The open-label design is a limitation of the study that affects mainly the safety assessment.

Conclusions: Women taking MQ IPTp (15 mg/kg) in the context of long-lasting insecticide treated nets had similar prevalence rates of low birth weight as those taking SP IPTp. MQ recipients had less clinical malaria than SP recipients, and the pregnancy outcomes and safety profile were similar. MQ had poorer tolerability even when splitting the dose over two days. These results do not support a change in the current IPTp policy.

Trial registration: ClinicalTrials.gov NCT00811421; Pan African Clinical Trials Registry PACTR 2010020001429343.

Trial 3

Intermittent preventive treatment of malaria in pregnancy with mefloquine in HIV-infected women receiving cotrimoxazole prophylaxis: a multicenter randomized placebo-controlled trial

doi: 10.1371/journal.pmed.1001735

Background: Intermittent preventive treatment in pregnancy (IPTp) with sulfadoxine-pyrimethamine (SP) is recommended for malaria prevention in HIV-negative pregnant women, but it is contraindicated in HIV-infected women taking daily cotrimoxazole prophylaxis (CTXp) because of potential added risk of adverse effects associated with taking two antifolate drugs simultaneously. We studied the safety and efficacy of mefloquine (MQ) in women receiving CTXp and long-lasting insecticide treated nets (LLITNs).

Methods and findings: A total of 1,071 HIV-infected women from Kenya, Mozambique, and Tanzania were randomized to receive either three doses of IPTp-MQ (15 mg/kg) or placebo given at least one month apart; all received CTXp and a LLITN. IPTp-MQ was associated with reduced rates of maternal parasitemia (risk ratio [RR], 0.47 [95% CI 0.27-0.82]; p=0.008), placental malaria (RR, 0.52 [95% CI 0.29-0.90]; p=0.021), and reduced incidence of non-obstetric hospital admissions (RR, 0.59 [95% CI 0.37-0.95]; p=0.031) in the intention to treat (ITT) analysis. There were no differences in the prevalence of adverse pregnancy outcomes between groups. Drug tolerability was poorer in the MQ group compared to the control group (29.6% referred dizziness and 23.9% vomiting after the first IPTp-MQ administration). HIV viral load at delivery was higher in the MQ group compared to the control group (p=0.048) in the ATP analysis. The frequency of perinatal mother to child transmission of HIV was increased in women who received MQ (RR, 1.95 [95% CI 1.14-3.33]; p=0.015). The main limitation of the latter finding relates to the exploratory nature of this part of the analysis.

Conclusions: An effective antimalarial added to CTXp and LLITNs in HIV-infected pregnant women can improve malaria prevention, as well as maternal health through reduction in hospital admissions. However, MQ was not well tolerated, limiting its potential for IPTp and indicating the need to find alternatives with better tolerability to reduce malaria in this particularly vulnerable group. MQ was associated with an increased risk of mother to child transmission of HIV, which warrants a better understanding of the pharmacological interactions between antimalarials and antiretroviral drugs.

Trial registration: ClinicalTrials.gov NCT00811421; Pan African Clinical Trials Registry PACTR 2010020001813440.

Table S1. Sensitivity multivariable analyses by age group: associations between gravidity and the study outcomes

Outcome	Age in categories					
	**≤ 19**		**20-24**		**≥ 25**	
	n=729*		*n*=628*		*n*=995*	
	Multivariable analyses – coefficients for gravidity**					
	OR (95% CI)	*p*-value	OR (95% CI)	*p*-value	OR (95% CI)	*p*-value
Low birth weight	0.40 (0.22-0.72)	<0.01	0.57 (0.26-1.24)	0.16	0.40 (0.10-1.62)	0.20
Small-for-gestational age	0.44 (0.29-0.68)	<0.001	0.79 (0.44-1.39)	0.41	1.49 (0.26-8.59)	0.66
Premature birth	0.70 (0.33-1.46)	0.34	0.46 (0.16-1.29)	0.14	0.28 (0.04-1.76)	0.17
Miscarriage	0.60 (0.09-3.92)	0.59	3.67 (0.21-64.51)	0.37	0.10 (0.02-0.61)	0.01
Stillbirth	1.40 (0.54-3.65)	0.49	0.37 (0.13-1.06)	0.06	0.17 (0.05-0.60)	<0.01
Hospital admissions***	1.01 (0.61-1.67)	0.96	0.95 (0.41-2.19)	0.91	-	-
Outpatient visits	1.07 (0.80-1.42)	0.66	0.90 (0.61-1.32)	0.59	0.89 (0.36-2.24)	0.81

*The n for each specific regression could be different from the one shown in the table due to missingness in the outcome variables

** Coefficients for multigravidae compared to primigravidae

*** Model for hospital admissions among women aged ≥ 25 years did not converge

Multivariable analyses adjusted for HIV status, study arm, gestational age at recruitment, MUAC at baseline, literacy and anaemia
